# Quasi-Solid-State Na–O_2_ Battery with Composite Polymer Electrolyte

**DOI:** 10.1021/acsami.4c04613

**Published:** 2024-07-02

**Authors:** Kevin Iputera, Cheng-Fu Tsai, Jheng-Yi Huang, Da-Hua Wei, Ru-Shi Liu

**Affiliations:** † Department of Chemistry, National Taiwan University, Taipei 106, Taiwan; ‡ Department of Mechanical Engineering and Institute of Manufacturing Technology, National Taipei University of Technology, Taipei 106, Taiwan; § Advanced Research Center For Green Materials Science and Technology, Taipei 106, Taiwan

**Keywords:** Na−O_2_ battery, quasi-solid-state, composite polymer electrolyte, oxygen reduction reaction, electrochemical reaction

## Abstract

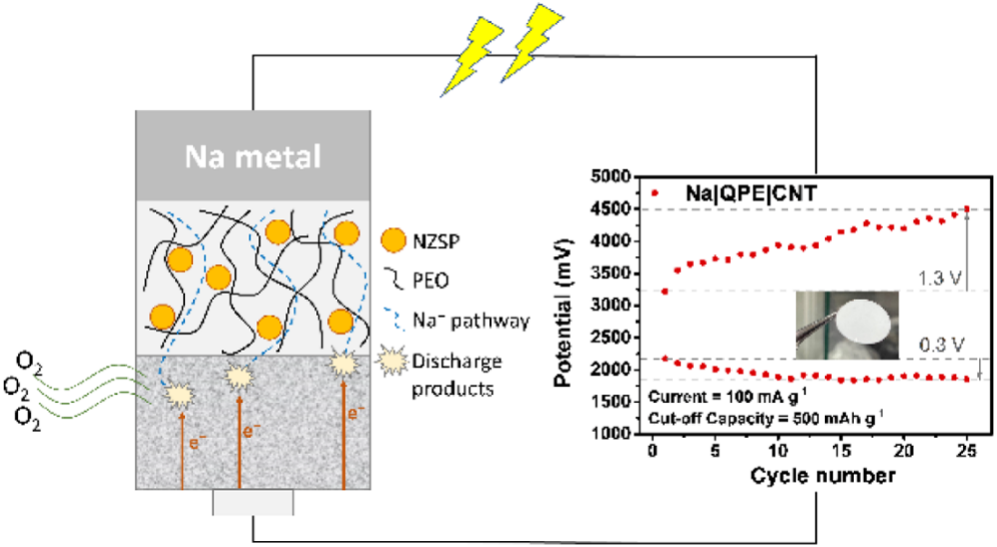

Na–O_2_ batteries have emerged as promising candidates due to their high theoretical energy density (1,601 Wh kg^–1^), the potential for high energy storage efficiency, and the abundance of sodium in the earth’s crust. Considering the safety issue, quasi-solid-state composite polymer electrolytes are among the promising solid-state electrolyte candidates. Their higher mechanical toughness provides superior resistance to dendritic penetration compared with traditional liquid electrolytes. The flexibility of the composite polymer electrolyte matrix allows it to conform to various battery configurations and considerably reduces safety concerns related to the combustion risks associated with conventional liquid electrolytes. In this study, we employed poly(ethylene oxide) (PEO) and sodium bis(trifluoromethanesulfonyl)imide (NaTFSI) as the polymer matrix and sodium ion-conducting agent, respectively. We incorporated nanosized NZSP (25 wt %) to create the composite polymer electrolyte membrane. This CPE design facilitates ion conduction pathways through both sodium salt and NZSP. By utilizing a liquid electrolyte infiltration method, we successfully enhanced its ionic conductivity, achieving an ionic conductivity of 10^–4^ S cm^–1^ at room temperature.

## 1 Introduction

Na–air batteries have become the focus of the development of next-generation energy storage.^1,2^ Compared with Li–air batteries, Na–air batteries introduce a Na metal anode to replace Li metal. This design can reduce the price of the battery, as Na is much more available than Li. The composition of discharge products may differ for different Na–O_2_ systems.^3^ Unlike Li–O_2_ batteries, which usually have Li_2_O_2_ as their main discharge product when using ether-based electrolytes, NaO_2_, other than Na_2_O_2_, could also be observed as the discharge product.^3,4^ NaO_2_ is kinetically stable and may not undergo disproportionation reaction to form Na_2_O_2_.^4^ Therefore, care should be exerted when analyzing the discharged cathodes of Na–O_2_ batteries to reveal their cell reaction. With certain cell conformation (differences in electrolyte and catalyst design), either NaO_2_ or Na_2_O_2_ could be the preferable discharge product.^3^ Whether NaO_2_ or Na_2_O_2_ dominates as the main discharge product depends on the stability of NaO_2_. If NaO_2_ cannot be stabilized, then Na_2_O_2_ would be dominant.

Hartmann et al.^4^ reported the clear formation of NaO_2_ supported by scanning electron microscopy (SEM) and X-ray diffraction (XRD) measurements. The distinct cubic crystal structure of NaO_2_ was depicted in the SEM images. Hartmann et al.^5^ later reported the low electronic conductivity of NaO_2_ as it is a wide-bandgap (2 eV) insulator, implying that the formation route passing NaO_2_ grains is not possible. In the same study, NaO_2_ was also found on the separator, showing the solubility of the O_2_^–^ species. If Na^+^ is solvated well in an electrolyte, O_2_^–^ will diffuse elsewhere, followed by nucleation and crystal growth.^6−8^ On the contrary, Liu et al.^9^ obtained Na_2_O_2_ as the main discharge product with evidence of selected-area electron diffraction (SAED). The principles mentioned above apply to quasi-solid-state and solid-state setups. When the content of the liquid electrolyte is decreased, the solution pathway is inevitably suppressed. In this case, the capacity is limited, and the overpotential increases as the kinetics of the cell reactions worsen. *In situ* TEM is also a powerful method to study the reaction of solid-state Na–O_2_ batteries.^10^ The nanobattery setup can visualize the microscopic changes in the cell reaction. Kwak et al.^11^ studied the reaction of carbon nanotubes (CNTs) via *in situ* TEM and obtained similar properties. NaO_2_ with a cubic morphology was identified, and Na_2_O_2_ formed as a film coating on the CNTs. These film-like products were a mixture of NaO_2_ and Na_2_O_2_ and were not fully decomposed during charge.

Recently, quasi-solid-state and solid-state metal–O_2_ batteries become more important due to the rising safety issue of Li-ion batteries.^12−14^ Nevertheless, the decrease in the liquid contents usually leads to a decrease in ionic conductivity and interfacial problems. Chang et al.^15^ prepared a quasi-solid-state Na–air battery with a sodium superionic conductor-type (NASICON-type) solid electrolyte. In their study, Na_3_Zr_2_Si_2_PO_12_ (NZSP) provided a high ionic conductivity of 2.7 × 10^–3^ S cm^–1^, while the electrode–NZSP interfaces were treated with a small amount of liquid electrolyte to improve the contact problem. Wang et al.^16^ developed a quasi-solid-state composite polymer electrolyte (QPE) that comprised PVDF-HFP, SiO_2_, and liquid electrolyte for Na–O_2_ battery, ensuring its conductivity as well as interfacial performance.

In this article, we introduce a design for quasi-solid-state Na–O_2_ batteries. A QPE is utilized as the separator and electrolyte. The design can reduce the contents of flammable liquid electrolytes in the system while providing acceptable ionic conductivity. Characterizations through SEM, energy-dispersive spectroscopy (EDS), and X-ray photoelectron spectroscopy (XPS) reveal the composition of the discharge products. The results are discussed with the knowledge we have of Na–O_2_ batteries in mind.

## 2 Experimental Section

### 2.1 Chemicals

Sodium carbonate (Na_2_CO_3_, 99.8%), zirconium dioxide (ZrO_2_, 99%), tetraethylene glycol dimethyl ether (TEGDME, 99%), and multiwalled carbon nanotubes (M.W.CNTs) (C, > 95%) were obtained from Sigma-Aldrich. Silicon dioxide (amorphous fumed) (SiO_2_, 99.99%) was obtained from Alfa Aesar. Ammonium dihydrogen phosphate (NH_4_H_2_PO_4_, 99%) was obtained from J. T. Baker. Ethanol (C_2_H_5_OH, 99.5%) was purchased from ECHO CHEMICAL CO. Ltd. Poly(ethylene oxide) (PEO, M.W. = 600,000) and polyvinylidene fluoride (PVDF, M.W. = 534,000) were purchased from Aldrich. Acetonitrile (CH_3_CN, 99.9%) was purchased from J. T. Baker. Sodium bis(trifluoromethanesulfonyl)imide (NaTFSI, 99.5%) was purchased from TCI. Sodium perchlorate (NaClO_4_, 99%) was purchased from Acros. *N*-Methyl-2-pyrrolidone (NMP, 99%) was obtained from Macron. NZSP pellets were purchased from China Glaze Co. Ltd.

### 2.2 NZSP Powder Preparation

NZSP was synthesized based on the solid-state synthesis method.^17^ The precursor weights of Na_3_Zr_2_Si_2_PO_12_ were calculated through a stoichiometric approach with a total precursor powder weight controlled at 3 g. The weights of Na_2_CO_3_, ZrO_2_, SiO_2_, and NH_4_H_2_PO_12_ were 0.7446 1.1541, 0.5625, and 0.5382 g, respectively. To avoid the formation of ZrO_2_ impurities in the final NZSP product due to ZrO_2_ from the ball-mill jar and grinding balls, the ZrO_2_ precursor weight was reduced by 20%. All of the precursors were placed in the milling jar, and ZrO_2_ balls were added at a weight ratio of 10:1 with respect to the powder. Ethanol was added as the wet milling agent and solvent for NH_4_H_2_PO_12_. The milling was carried out at a speed of 300 rpm for 24 h to achieve a homogeneous mixture of all the precursors. The high-energy milling process also enabled a preliminary phase formation. After milling, the homogeneous precursor slurry was collected, and the ethanol was removed through evaporation. The resulting precursor powder was then loaded into an Al_2_O_3_ crucible, and the synthesis of the NaSICON phase was carried out in an atmospheric environment at a temperature of 1150 °C for 5 h using a square furnace. After being sintered, NZSP formed highly agglomerated ceramic pellets. Wet milling was performed with ethanol as the milling agent at a speed of 450 rpm for 5 h to grind the agglomerated pellets into nanosized powder, thus completing the synthesis of nanosized NZSP ceramic powder.

### 2.3 CPE/QPE Preparation

PEO was used as the main component and dissolved in acetonitrile as the solvent to form a slurry. Sodium electrolyte salt, NaTFSI, was added as the main ion conductor, and nanosized NZSP was incorporated as an active filler. The PEO-to-NaTFSI ratio was fixed at a molar ratio of 15:1, and the addition of NZSP ranged from 10 to 30 wt %. It should be noted that the molar ratio of PEO was counted by its monomer rather than as a whole. The slurry was poured onto a PTFE substrate and dried in an argon-filled glovebox at 50 °C. The use of a PTFE substrate during the preparation of the polymer electrolyte membrane helps prevent the film from sticking and facilitates easy demolding. The preparation process was conducted in an argon-filled glovebox to prevent contamination of the samples from contact with moisture and oxygen. After complete evaporation of the ACN solvent, the film was cut into suitable sizes for later applications.

### 2.4 Air Cathode Preparation

The cathode utilized CNT as both the electrical conductor and substrate for the discharge products to settle. A slurry was prepared by mixing CNT with PVDF as the binder and NMP as the solvent. The weight ratio of CNT to PVDF was 9:1 to ensure sufficient adhesion. 180 mg of CNT, 20 mg of PVDF, and 9 mL of NMP were mixed using a stirrer for at least 24 h to obtain a homogeneous cathode slurry. Carbon paper was cut with a diameter of 13 mm and was washed with acetone to remove surface contaminants. The cleaned carbon paper was placed in an 80 °C oven to remove moisture and residual solvent. This treated carbon paper served as the cathode substrate (current collector). 5 μL of the slurry was extracted using a micropipette and drop-casted onto the carbon paper. The coating was spread evenly from the center of the carbon paper, with the aim of a thin and uniform layer. After coating, the cathode was dried in a vacuum oven at 80 °C for 1 day. The weight of the dried cathode was measured, and the weight of the carbon paper was subtracted to determine the weight of the cathode material. The weight of active materials ranged from 0.2–0.5 mg per cathode. The cathode was then ready for the battery assembly and discharge testing.

### 2.5 Battery Assembly

2032-type coin cell was used for battery tests and was assembled in a glovebox under an Ar environment. First, the cathode with the coated side facing toward the separator was placed in the center of the bottom cover of the button cell, followed by placing the QPE as the separator/ion-conductive electrolyte. A 10 mm diameter sodium metal disk was placed above the QPE as the anode. Finally, a metal plate spring was placed on top, and the battery was sealed with the top cover using a specialized sealing device, completing the battery assembly. The battery was then placed in a custom-made testing vessel. Pure oxygen (purity = 99.9%) gas was introduced into the chamber through one inlet, while the air inside the chamber was vented through another outlet. The oxygen gas was pressurized to 5 psi, and the filling process was completed within 5 min. The input oxygen was 50 times more than the volume of the vessel, ensuring the complete removal of residual air. After filling, the battery system was left to stand for 8 h to allow for stable diffusion of gases within the chamber. Once the battery system reached a steady state, it was ready for electrochemical measurements.

### 2.6 Characterization Methods

In this study, several instruments were employed to characterize and analyze the materials and electrochemical systems. X-ray diffraction (XRD) was conducted using a Bruker D2 phaser diffractometer to investigate the crystallographic properties of the samples. The multichannel potentiostat/galvanostat, Autolab PGSTAT302N from Metrohm, was utilized to measure the electrochemical impedance spectra of the quasi-solid-state polymer electrolytes (CPEs). CHI611E potentiostat/galvanostat from CHI Instruments was utilized for cyclic voltammetry (CV) measurements. Scanning electron microscopy (SEM) imaging of the oxygen battery discharge products was performed using a ZEISS ΣIGMA Essential SEM. Furthermore, charge–discharge tests on the sodium–oxygen battery were conducted by using a BAT-750B cycling machine from AcuTech. These instruments provided valuable insights into the structural, electrochemical, and morphological characteristics of the studied materials and electrochemical systems.

## 3 Results and Discussion

The structure of NZSP can be described as a *P*_1_ crystal structure as shown in Figure. S1, indicating that the synthesized NZSP was pure without ZrO_2_ impurities, as ZrO_2_ presented in the commercial pellet. In accordance with the synthesis method of NZSP described in the experimental section, we synthesized NZSP on the basis of chemical stoichiometry but failed to obtain a pure phase with ZrO_2_ impurities. We resolved the issue by decreasing the ZrO_2_ content. We hypothesized that the extra ZrO_2_ in the synthesized powders originated from peeling off the ZrO_2_ grinding balls. The amount of ZrO_2_ was thus reduced by 20% on the basis of its stoichiometric value. The synthesized NZSP powders were of a submicron size, as depicted in the result of dynamic light scattering (DLS; Figure S2), with an average particle size of 302 nm. The DLS result can be double-checked by the SEM result in Figure S3. The secondary particle size was submicron and close to the particle size obtained by DLS. The primary particle size was tens of nanometers. The consistency between the particle size obtained from DLS and the secondary particle size obtained from SEM implies that the secondary particles were difficult to separate. Given that we failed to break aggregated particles in the DLS measurement via supersonication, we suppose that the NZSP particles in the CPE were mostly secondary. Nevertheless, the nanosized particles can increase the conductivity of the CPE due to the high contact area between NZSP and PEO.^18^

**Figure 1:**
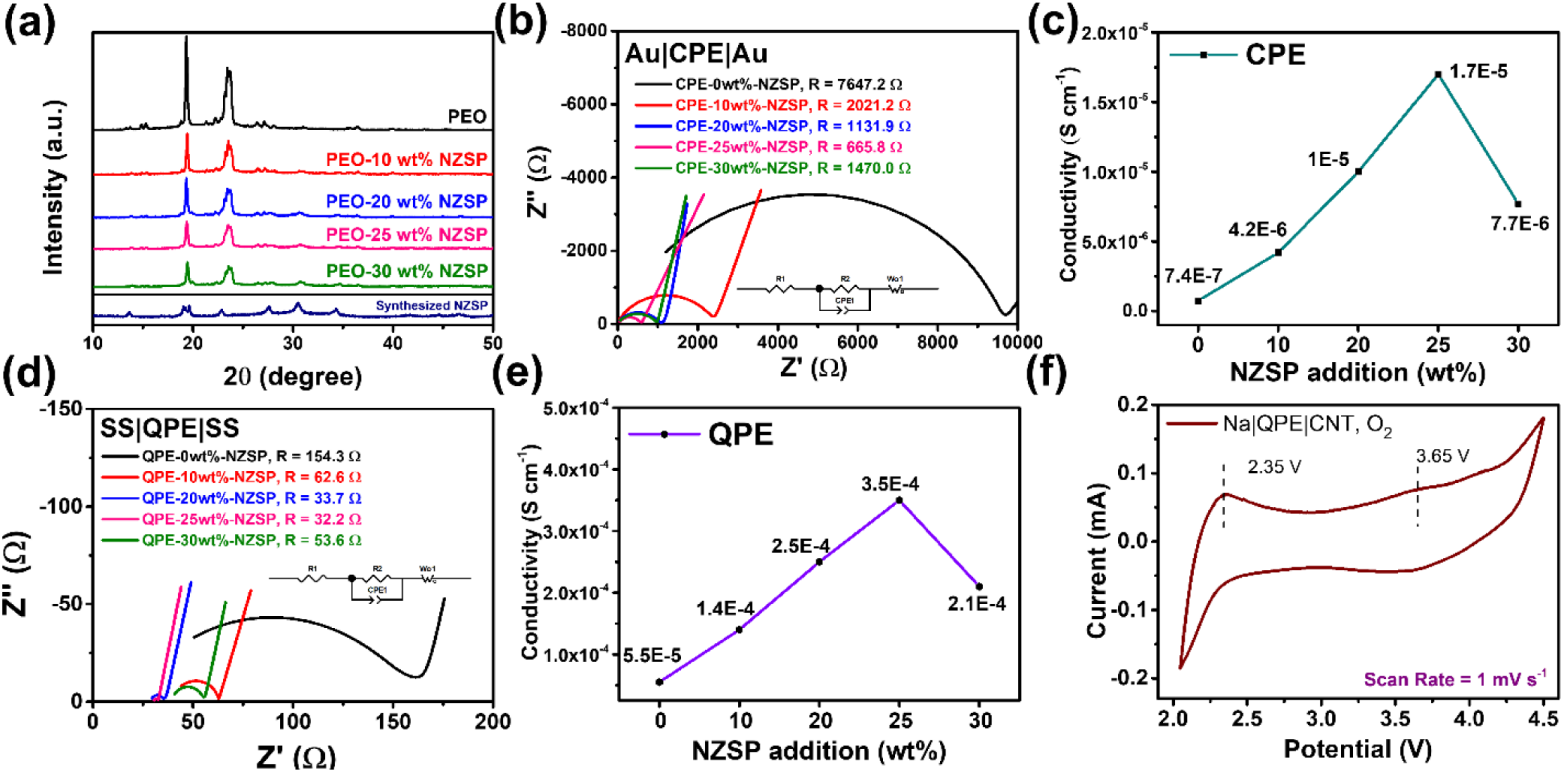
(a) XRD patterns, (b) EIS measurement, and (c) calculated conductivities of CPE with different NZSP contents. (d) EIS measurement, and (e) calculated conductivities of QPE with different NZSP contents. (f) CV profile of Na–O_2_ battery with QPE as electrolyte.

The prepared NZSP powders were pelletized to measure the conductivity. As shown in Figure S4, the total conductivity was calculated to be 1.1 × 10^–4^ S cm^–1^. The total conductivity was slightly lower than the bulk conductivity (3.8 × 10^–4^ S cm^–1^) because the grain boundary generates severe resistance in pellets.^17^ However, the observed value at the scale of 10^–4^ S cm^–1^ is consistent with that in previous studies.^17,19^ The conductivity of the NZSP pellets was 1 order higher than that of their CPE counterpart. However, we did not consider using NZSP pellets in our study because of their thickness. Although the thickness of NZSP can be reduced by polishing the pellet, the pellet is brittle and breaks easily during the battery assembly. Moreover, the thick NZSP pellet inevitably reduces the gravimetric and volumetric energy densities. Thus, we prepared CPEs with different compositions and checked their properties. As shown in Figure 1a, the XRD peaks attributed to PEO could be clearly observed in all the samples. Crystallinity decreased when NZSP was introduced, but the effect was not obvious. We then measured the conductivities and found that CPE-25 wt %-NZSP had the highest ionic conductivity (Figure 1b,c). The activation energies (*E*_a_) were also measured, and CPE-25 wt %-NZSP had the lowest *E*_a_, which is in accordance with the conductivity tests (Figure S5). The ionic conductivity was high at high temperatures for all of the samples. We conducted a study on the Na–O_2_ battery at room temperature due to the high reactivity of Na metal.^20^ The reason is that Na metal undergoes severe degradation at high temperatures.^20,21^ We assembled all-solid-state Na–O_2_ batteries with CPE-25 wt %-NZSP. However, we failed to obtain sustainable cycle performance and good full-discharge capacity. We assume that the poor performance originated from the poor interfaces between the CPE and both electrodes.

To address this problem, we introduced liquid electrolytes to the CPEs and turned them into QPEs. The original intention of using solid-state electrolytes was to reduce the flammability of batteries. The safety issue can be addressed by reducing the content of liquid electrolytes in the battery system. Therefore, we used QPEs instead of CPEs to obtain a sustainable electrochemical performance. QPE was prepared by soaking CPE in liquid electrolyte (1 M NaTFSI in TEGDME) overnight (Figure S6). NaTFSI was used in the liquid electrolyte due to the dual-salt effect, as reported previously.^22,23^ The trend of ionic conductivity followed that of CPEs, as shown in Figure 1d,e, with QPE-25 wt %-NZSP possessing the highest ionic conductivity. The ionic conductivity of 3.5 × 10^–4^ S cm^–1^ was now comparable to that of the NZSP pellet; therefore, we considered the ionic conductivity to be sufficient for our study. Before we examined the performance of the battery, we performed one last check on the stability of QPE. The charge potential of a common Na–O_2_ battery can be as high as 4.5 V, and conventional liquid electrolytes for Na–O_2_ batteries (i.e., TEGDME) will decompose at such a high oxidation potential. Nevertheless, we observed that the oxidative current increased only after 4.8 V. The improved oxidation stability could be attributed to the low content of TEGDME and the stable CPE, as shown in Figure S7.

The cyclic voltammetry (CV) profiles of QPE are shown in Figure 1f. The first feature addressed was the reversible redox at 2.0–2.5 V. This redox was attributed to the O_2_/O_2_^–^ couple.^24^ Unlike the superoxide species in Li–O_2_ batteries, NaO_2_ can be stable in Na–O_2_ batteries.^3^ The other oxidation peak at 3.65 V was attributed to an irreversible oxidation reaction. The irreversibility can be traced back to the extra chemical reaction of NaO_2_ to Na_2_O_2_. The disproportionation reaction cannot convert the Gibbs free energy to electric energy, which means that the energy harvested during discharge is inevitably lower than that applied during charge (even when the reactions are totally thermodynamically reversible). Although the thermodynamically favored disproportionation reaction is difficult to prevent, a comparable current can still be obtained for the reversible reversible O_2_/O_2_^–^ redox reaction.

**Figure 2:**
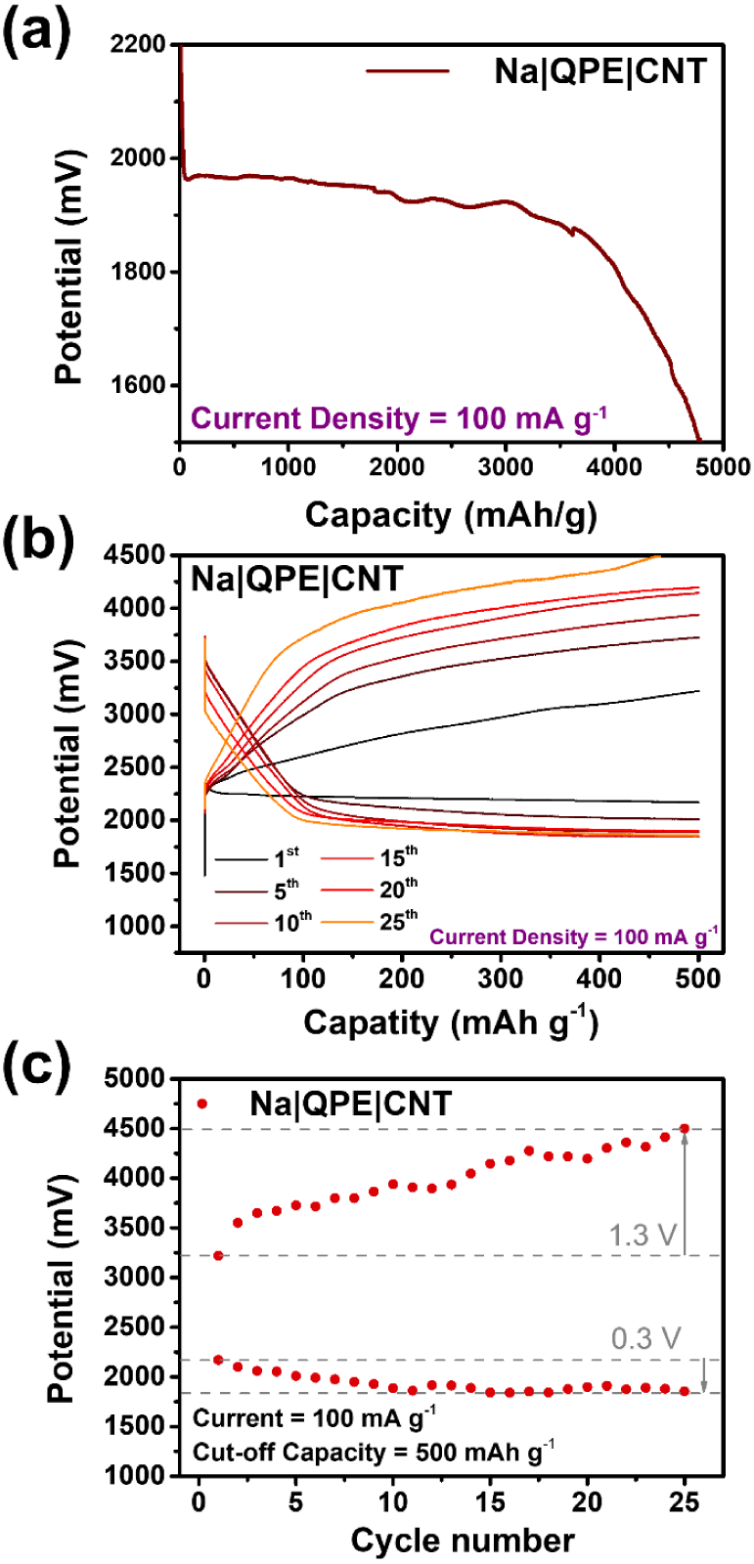
(a) Full discharge and (b) cycle test of quasi-solid-state Na–O_2_ battery with QPE-25 wt %-NZSP as electrolyte. The current density was set as 100 mA g^–1^ and a cutoff capacity of 500 mAh g^–1^ was set for cycle tests. (c) Potential change over cycles. The potential value was that at cutoff capacity.

The symmetric cell, Li|QPE(QPE-25 wt %-NZSP)|Li, showed good stability with a cycle duration of around 250 h, as shown in Figure S8. A full-discharge test was conducted on a quasi-solid-state Na–O_2_ battery with QPE (Figure 2a). The cell was discharged at a current density of 100 mA g^–1^ and had a capacity of around 4,800 mAh g^–1^. The obtained capacity was comparable to that in previous reports, implying a sufficient number of active sites.^25^ Next, we conducted cycle tests at the same current density of 100 mA g^–1^ and a cutoff capacity of 500 mAh g^–1^ (Figure 2b). Figure 2c indicates that the charge–discharge potential gap was small in the beginning but gradually increased over the cycles. Notably, the charge overpotential increased more rapidly compared to its discharge counterpart. The charge overpotential increased by 1.3 V after 25 cycles, whereas the discharge overpotential increased by only 0.3 V. The rapid increase in charge (over)potential implies that the main component of the discharge products changed from NaO_2_ to Na_2_O_2_, which is difficult to decompose. To rationalize this observation, we propose that NaO_2_ is disproportionate to form Na_2_O_2_ over time, resulting in a constant change in the composition of the discharge product. The NaO_2_ that turned to Na_2_O_2_ may have been unreachable by the cathode or simply reacted before it could be oxidized electrochemically. Meanwhile, the discharge platform did not decrease dramatically over the cycles. This phenomenon can be explained by the relatively easy-to-excess reactants. Although we observed some left-over discharge products that were not decomposed during charge, they did not block the active sites because the good solubility of the intermediate/discharge product may have diffused and settled elsewhere.^5^

**Figure 3:**
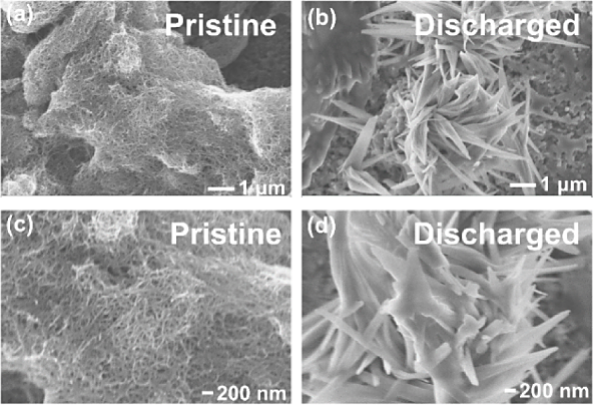
SEM images of (a) pristine and (b) discharged cathode. (c) and (d) are the enlarged images of (a) and (b), respectively. The discharged cathode was discharged to 3000 mAh g^–1^ at a current density of 100 mA g^–1^.

Through the hypothesis proposed above, we provide evidence to support our statements. The SEM images in Figure 3 show that spike-like discharge products were formed in the filamentous CNT structure. The morphology did not clearly indicate whether it was NaO_2_ or other discharge products since NaO_2_ gives the cubic structure while Na_2_O_2_ gives the hexagonal one. The cubic structure of NaO_2_ can be observed easily, as reported by Bender et al.^26^ Kwak et al.^11^ found the morphology of the mixture of NaO_2_ and Na_2_O_2_ being film-like structures using *in situ* TEM. Therefore, the reduction reaction leads to the formation of a mixture of various discharge products rather than a single product. We conducted EDS measurements on the discharged and charged air cathodes in Figures S9 and S10. Although EDS is not an accurate method to determine the elemental composition, EDS analysis on the discharged products (middle part of the SEM image in Figure S9) shows the Na:O ratio being 1:2.5, proving that the oxygen contents are high on the surface. The reduction of the ratio of O and Na supports the decomposition of the main part of the discharge products. Still, some discharge products remained since residue O and Na gave a considerable amount in the EDS analysis. The large, spike-like products were removed after being charged, but the filamentous structure of CNT was no longer observed. Thus, we assumed that the discharge products that formed as a film covering CNT were not decomposed.^11^

**Figure 4:**
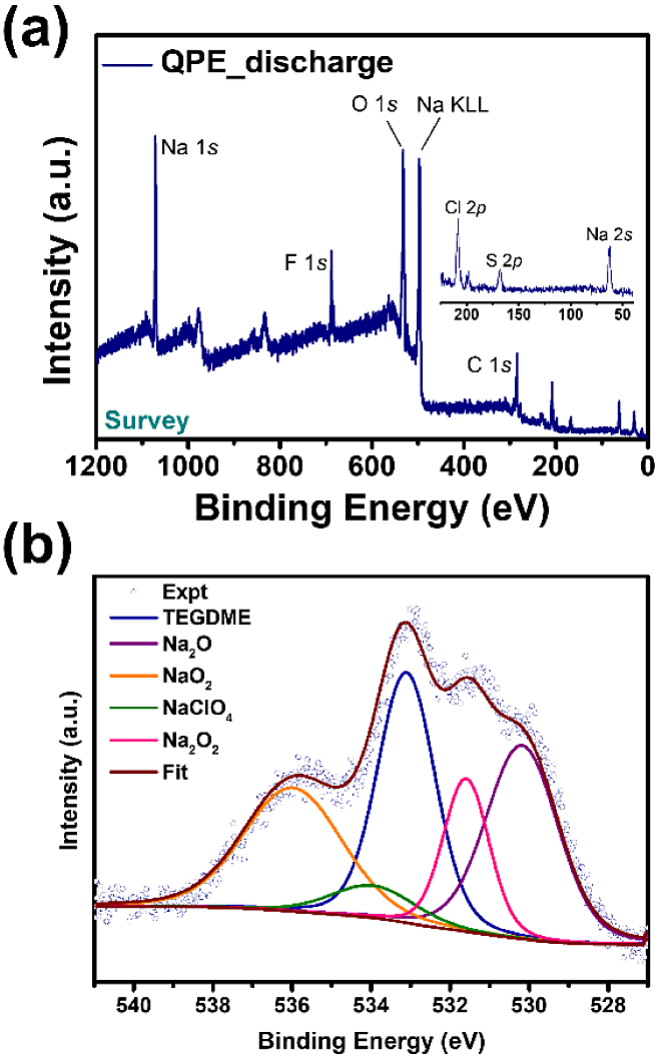
(a) Survey and (b) O 1s XPS spectrum of the discharged cathode.

XPS was conducted to reveal the composition of the discharge products, because XPS is a powerful tool for determining the oxidation states of elements. The survey scan showed the presence of elements, all of which could be assigned to known components in batteries (Figure 4a,b). We focused on the O 1*s* spectrum, because it can provide valuable information. As shown in Figure 4b, Some signals should be attributed to NaTFSI, but they were too small for us to clearly observe because we did not observe clear S 2*p* and N 1*s* (Figure 4a). The signals of NaClO_4_ and the decomposition product of TEGDME were assigned at 534 and 533.1 eV, respectively.^27−29^ We propose that Na_2_O (530.1 eV),^30^ Na_2_O_2_ (531.6 eV),^31^ and NaO_2_ (536 eV)^32^ served as the discharge product of our quasi-solid-state Na–O_2_ battery. The formation of NaO_2_ was supported by CV, as mentioned previously. Moreover, EDS analysis (Figure S9) of the discharge products also showed high oxygen contents, supporting the presence of NaO_2_ and Na_2_O_2_ on the surface. The formation of Na_2_O and Na_2_O_2_ could have been the result of disproportionation reactions. We could not eliminate the possibility of the direct reduction to Na_2_O and Na_2_O_2_, but such a possibility is low because of the quasi-solid-state electrolyte and the limited ionic and electronic conductivity of Na_2_O and Na_2_O_2_. The former restricts the solution reaction pathway and may block the active sites, and the latter limits the reaction interface to QPE|CNT.

## 4 Conclusions

We successfully developed a quasi-solid-state Na–O_2_ battery by using QPE. We utilized poly(ethylene oxide) as the polymer matrix and incorporated sodium electrolyte salt NaTFSI and nanosized sodium superionic conductor NZSP powder to modulate the electrochemical impedance and ion conduction of the polymer thin film. Subsequently, a quasi-solid-state polymer electrolyte was prepared by using a trace electrolyte infiltration method and successfully applied in Na–O_2_ batteries. The quasi-solid-state composite with a trace electrolyte (QPE-25 wt %-NZSP) showed a conductivity of 3.5 × 10^–4^ S cm^–1^ at room temperature. Moreover, QPE-25 wt %-NZSP exhibited a wide electrochemical stability window of 5.0 V, which matches the high charge potential of the Na–O_2_ battery. The QPE-based sodium–oxygen battery achieved a high discharge capacity of 4789 mAh g^–1^ at a current density of 100 mA g^–1^ in the absence of catalysts. It demonstrated a cycling performance of 25 cycles with a cutoff capacity of 500 mAh g^–1^. These findings demonstrate the feasibility of applying quasi-solid-state polymer electrolytes to Na–O_2_ batteries. These electrolytes offer a safe, efficient alternative with a low liquid electrolyte content.

## Supporting Information Available

Figures S1–10, additional materials, characterization, and electrochemical data (PDF)
