# Supplementary material for: Quasi-Solid-State Na–O2 Battery with Composite Polymer Electrolyte
Source: ACS Appl Mater Interfaces. 2024 Jul 2;16(28):36289–94. doi: 10.1021/acsami.4c04613 (PMC11261556; doi:10.1021/acsami.4c04613)
Supplement: Supplementary file 1 — am4c04613_si_001.pdf [file am4c04613_si_001.pdf]

Supporting Information

Quasi-solid-state $Na–O_2$ Battery with Composite Polymer Electrolyte

Kevin Iputera,[a,‡] Cheng-Fu Tsai,[b,‡] Jheng-Yi Huang,[a] Da-Hua Wei,[b,*] and Ru-Shi Liu[a,c*]

[a]Department of Chemistry, National Taiwan University, Taipei 106, Taiwan.

[b]Department of Mechanical Engineering and Institute of Manufacturing Technology, National Taipei University of Technology, Taipei 106, Taiwan

[c]Advanced Research Center For Green Materials Science and Technology, Taipei 106, Taiwan.

* Corresponding authors

Correspondence and requests for materials should be addressed to Da-Hua Wei (email: dhwei@ntut.edu.tw) or Ru-Shi Liu (email: rsliu@ntu.edu.tw).

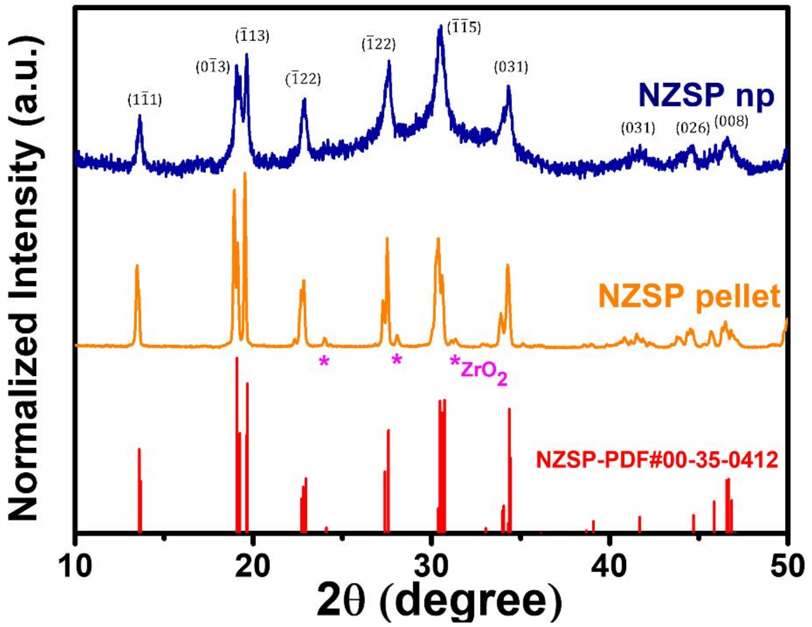

Figure S1. XRD patterns of NZSP nano-powders and commercial NZSP pellets.

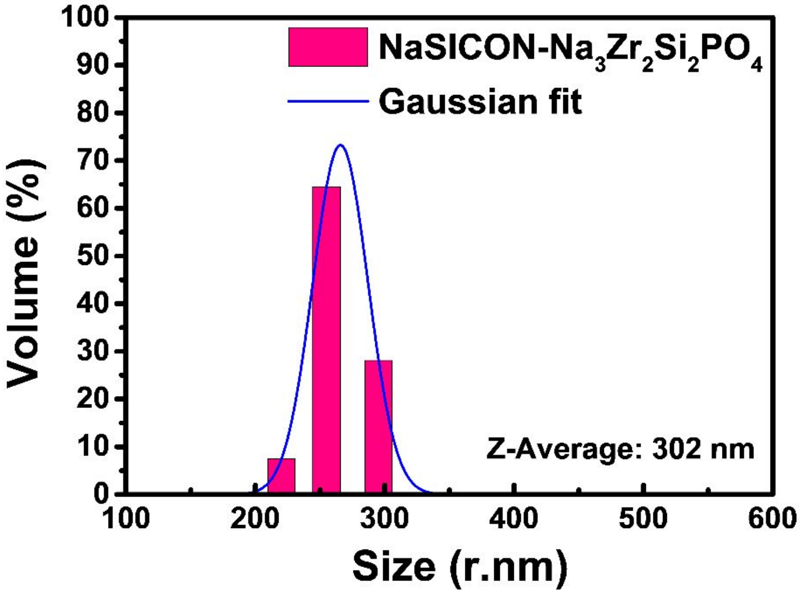

Figure S2. DLS analysis of NZSP nano-powders. The powders were dispersed in ethanol and super-sonicated before measurement.

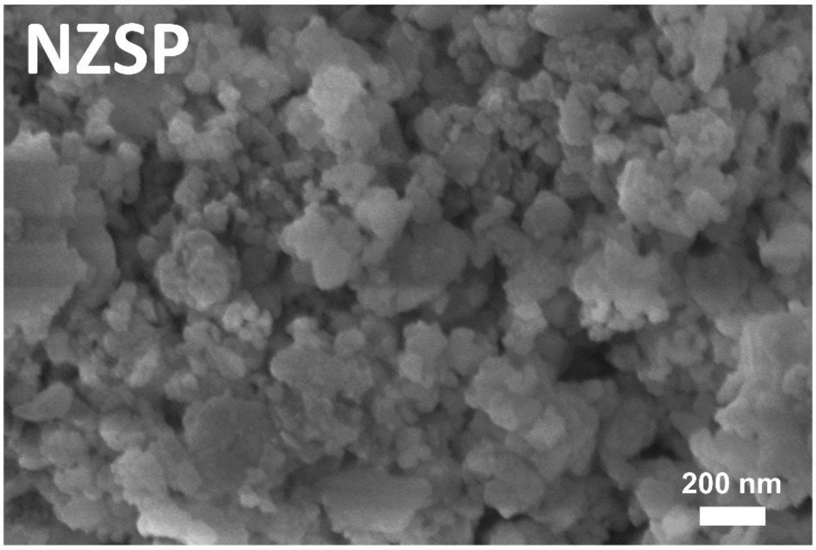

Figure S3. SEM image of the synthesized NZSP nano-powders.

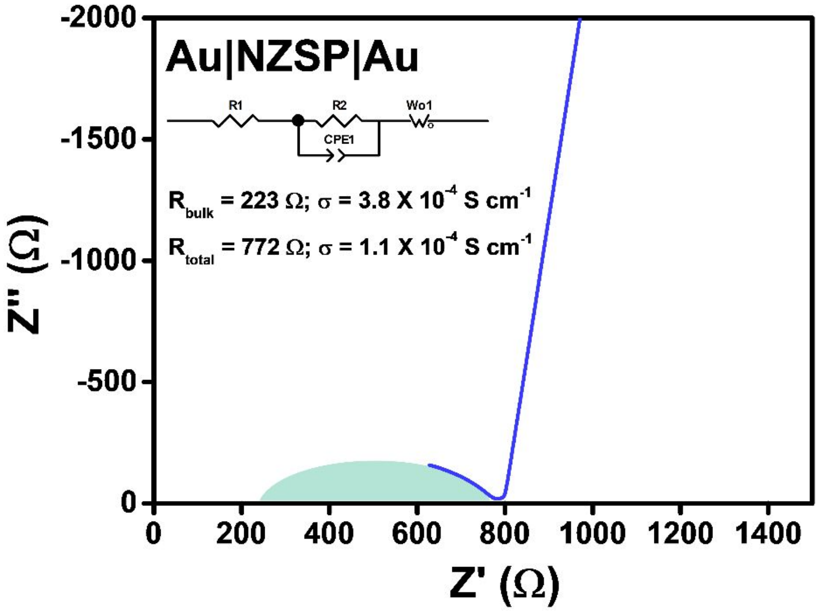

Figure S4. EIS measurement of cold-pressed NZSP pellet with synthesized NZSP nano-powder.

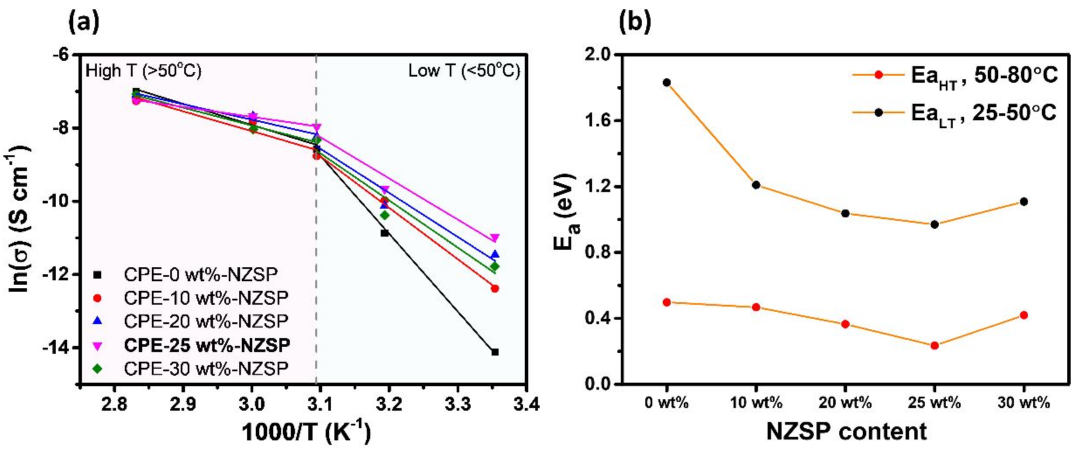

Figure S5. (a) Arrhenius plot of CPE with different NZSP contents. (b) Calculated value of activation energy at high and low temperature regions.

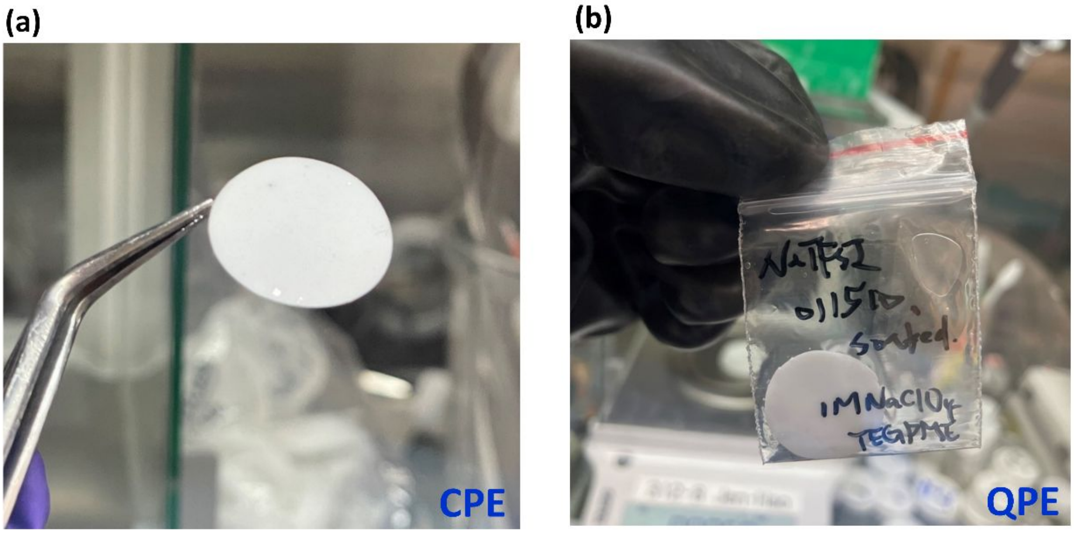

Figure S6. Photos of (a) CPE and (b) QPE. The latter was soaked in 1 M $NaClO_4$/TEGDME overnight.

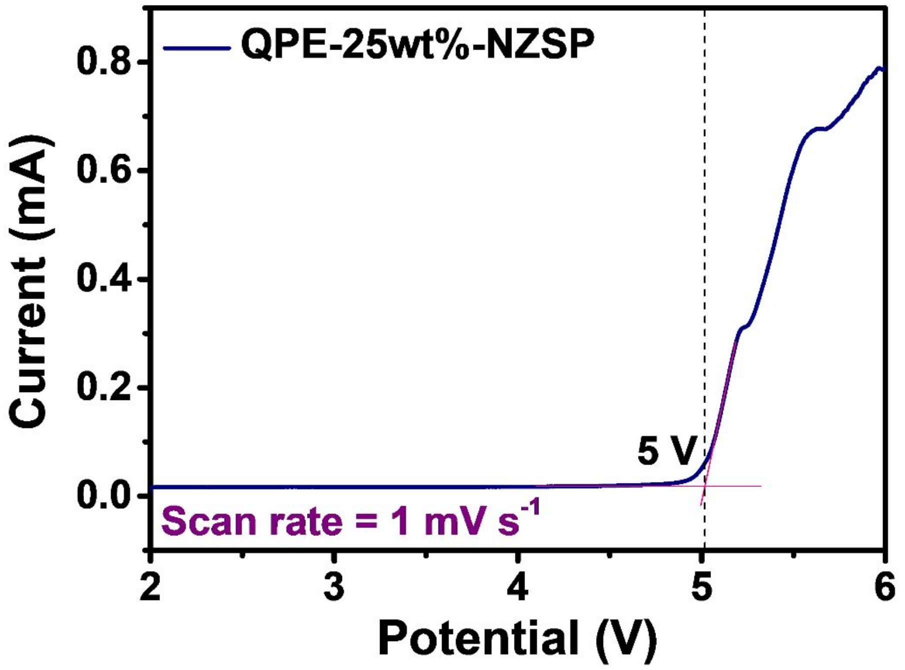

Figure S7. LSV curve of the Na|QPE|SS cell. The stability of QPE was up to 5 V.

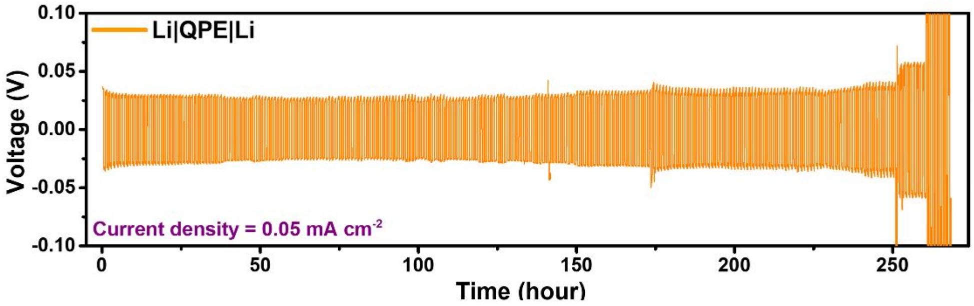

Figure S8. Li|QPE|Li symmetric cell test with a current density of 0.05 mA cm^{-2} and a cutoff capacity of 0.025 mAh cm^{-2}.

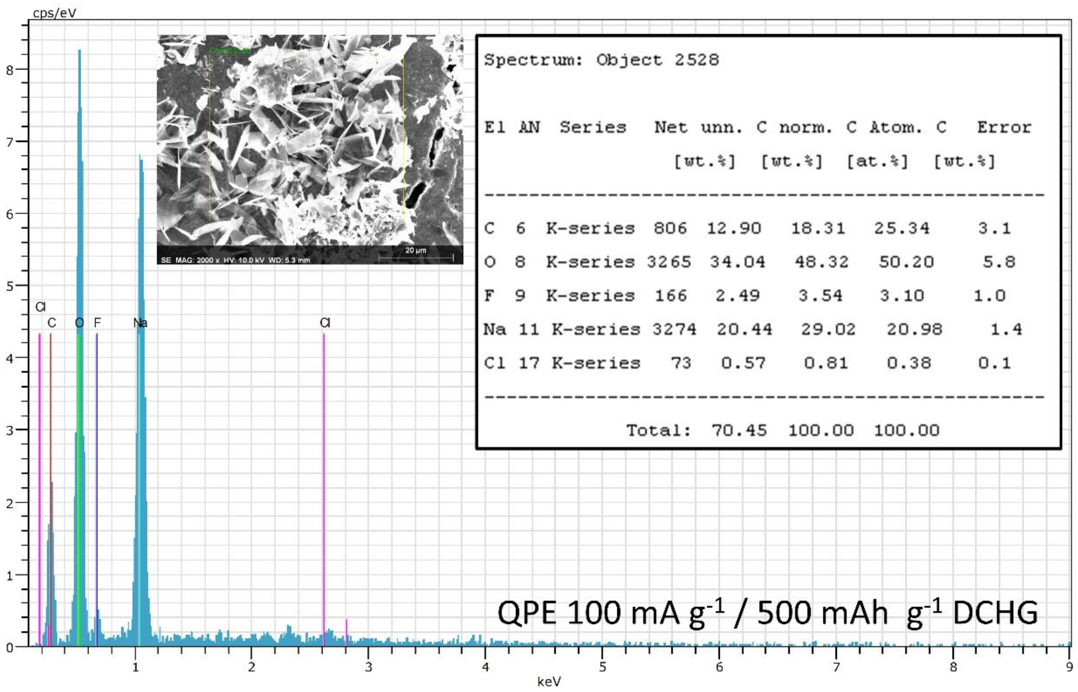

Figure S9. EDS analysis on the discharged cathode. The cathode was discharged to a capacity of 500 mAh g^{-1} at a current density of 100 mA g^{-1}.

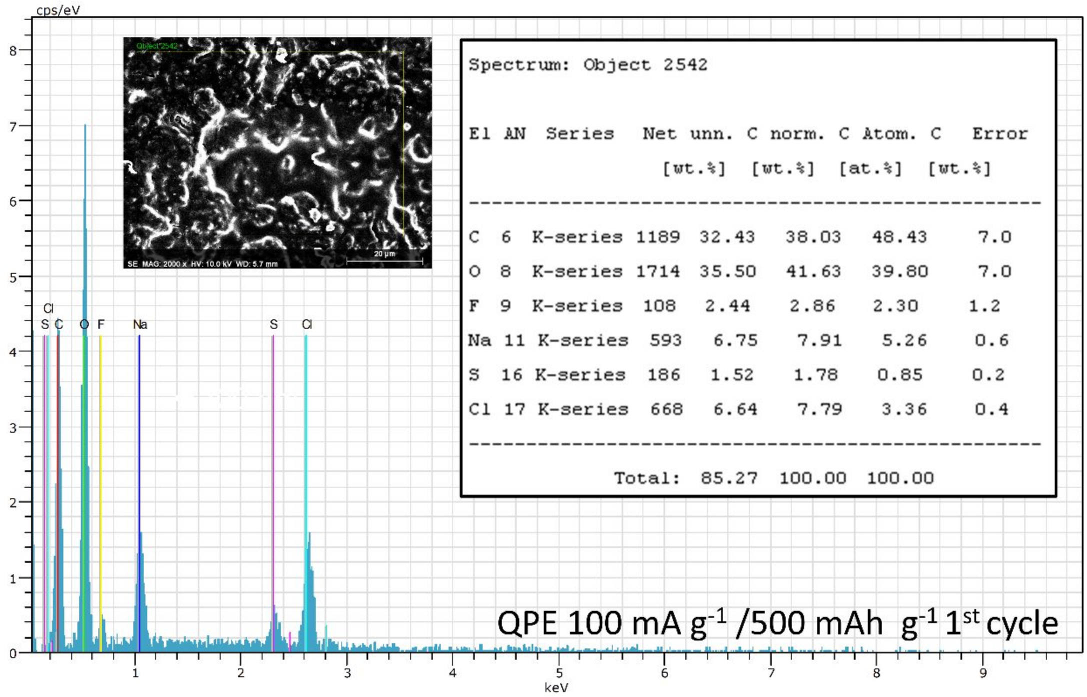

Figure S10. EDS analysis on the recharged cathode at the first cycle. The cathode was recharged to a capacity of 500 mAh g^{-1} at a current density of 100 mA g^{-1}.
